# Genomic data as the “hitchhiker's guide” to cattle adaptation: tracking the milestones of past selection in the bovine genome

**DOI:** 10.3389/fgene.2015.00036

**Published:** 2015-02-10

**Authors:** Yuri T. Utsunomiya, Ana M. Pérez O'Brien, Tad S. Sonstegard, Johann Sölkner, José F. Garcia

**Affiliations:** ^1^Departamento de Medicina Veterinária Preventiva e Reprodução Animal, Faculdade de Ciências Agrárias e Veterinárias, Universidade Estadual Paulista (UNESP)Jaboticabal, São Paulo, Brazil; ^2^Division of Livestock Sciences, Department of Sustainable Agricultural Systems, University of Natural Resources and Life Sciences (BOKU)Vienna, Austria; ^3^Animal Genomics and Improvement Laboratory, Agricultural Research Service, United States Department of AgricultureBeltsville, MA, USA; ^4^Laboratório de Bioquímica e Biologia Molecular Animal, Departamento de Apoio, Saúde e Produção Animal, Faculdade de Medicina Veterinária de Araçatuba, Universidade Estadual Paulista (UNESP)Araçatuba, São Paulo, Brazil

**Keywords:** positive selection, *Bos taurus*, *Bos indicus*, adaptation, single nucleotide polymorphism

## Abstract

The bovine species have witnessed and played a major role in the drastic socio-economical changes that shaped our culture over the last 10,000 years. During this journey, cattle “hitchhiked” on human development and colonized the world, facing strong selective pressures such as dramatic environmental changes and disease challenge. Consequently, hundreds of specialized cattle breeds emerged and spread around the globe, making up a rich spectrum of genomic resources. Their DNA still carry the scars left from adapting to this wide range of conditions, and we are now empowered with data and analytical tools to track the milestones of past selection in their genomes. In this review paper, we provide a summary of the reconstructed demographic events that shaped cattle diversity, offer a critical synthesis of popular methodologies applied to the search for signatures of selection (SS) in genomic data, and give examples of recent SS studies in cattle. Then, we outline the potential and challenges of the application of SS analysis in cattle, and discuss the future directions in this field.

## Introduction

The ability of domestic cattle to convert low-quality forage into meat, milk, and draft power is of direct importance to the livelihood of the human species. This ability is tightly linked to the adaptation of indigenous and exotic cattle to the diverse environments found around the world, which may result from complex—mostly untold—stories of migration, expansion, exposure to diseases, admixture, climate changes, and selective pressures (Ajmone-Marsan, 2010). These past events have shaped the genetic diversity of domestic cattle throughout history, and their present genomes may shelter tractable signatures of these phenomena.

Footprints of selection, such as specific patterns of change in allele frequencies, diversity loss, and haplotype structure, are currently detectable from single nucleotide polymorphism (SNP) data by well-established methodologies (Sabeti et al., 2006; Oleksyk et al., 2010), and can unravel past responses of the cattle genome to natural and human-driven selection, as well as evidences of loci and variants underlying adaptive and economically important traits. Detecting these selection signatures (SS) may not only help to shed some light on the key adaptive events that have generated the enormous phenotypic variation observed between cattle breeds today, but can also be of biotechnological relevance.

In recent years, SS studies are becoming increasingly popular because they offer a complementary strategy to genome-wide association (GWA) studies on mapping variants impacting traits of interest, helping to link phenotypes to gene function. In a typical GWA analysis, one starts from a phenotype and scan genotypes to identify underlying large and moderate effect variants (Bush and Moore, 2012). Generally, SS studies take the opposite direction: one starts with an evidence of selection in samples sharing geographical proximity, environmental factors or a common phenotype, and attempts to find selected mutations (Sabeti et al., 2006).

The major motivation for SS studies is that this type of approach can pinpoint chromosomal segments sheltering large effect mutations even if they no longer segregate in a population. In such cases, these variants cannot be detected by classical quantitative genetics methods unless linkage experiments are designed using crosses (Ramey et al., 2013). Another appealing feature of SS studies is that they typically require over 10 fold smaller sample sizes in comparison to GWA studies. Moreover, SS can reveal signals on genes controlling traits that are difficult, expensive or even impossible to measure on a large population (i.e., disease resistance).

Although identifying SS is of paramount importance to uncover variants responsible for adaptive traits, its application to cattle data must be carefully interpreted as important demographic events such as severe population bottlenecks, genetic drift, and admixture, as well as confounding effects derived from the development of SNP panels, may give rise to false signals. Furthermore, SS have serious limitations in targeting specific traits, so assigning signals to phenotypes is a non-trivial problem.

Here, we provide a brief review of the demographic history of cattle as it is known, present a critical review of some of the main methodologies available for the detection of putative loci under selection, and provide examples of recently published results in cattle. Then, we outline the potential and the challenges of the application of these methods to cattle data, and discuss the future directions in this field.

## Cattle demographic history

### Differentiation and domestication

The humpless taurine (*Bos taurus*) and the humped indicine or zebu cattle (*Bos indicus*) descend from the wild ox or auroch (*Bos primigenius*), which has been extinct since 1627 (Mona et al., 2010). The two populations of wild aurochsen that formed the ancestral pool for these interfertile cattle species may have diverged some 280,000 years ago (Murray et al., 2010), and were subjected to many demographic events before being domesticated by our species, including severe bottlenecks, admixture and natural selection.

Although the domestication of taurine and zebu cattle is still an open question and an active field of investigation, evidences collected over 20 years of research on molecular genetic diversity of cattle (see Groeneveld et al., 2010, for further review), combined with historic and archaeological data, support that these species were independently domesticated in at least two episodes (Loftus et al., 1994; Bruford et al., 2003): some 10,000 years ago, taurine animals were captured from the wild in the Fertile Crescent (modern-day countries of Israel, Jordan, Lebanon, Cyprus and Syria, and parts from Egypt, Turkey, Iraq, Iran, and Kuwait); 1500 years later, zebu cattle were domesticated in the Indus valley (present Northeastern Afghanistan, Pakistan, and Northwestern India).

### Expansion

Taurine cattle have an almost cosmopolitan distribution today. From the domestication centre in Southwestern Asia, they followed human migrations and slowly expanded over Asia and Europe. An independent domestication episode of taurine cattle in Northeastern Africa is disputed, but as molecular data are not conclusive, the divergence of African and Eurasian taurine populations from a common ancestor domesticated in the Fertile Crescent is well supported (Hanotte et al., 2002; Ajmone-Marsan, 2010; Decker et al., 2014). Other isolated micro-events of domestication are also not discarded, not even interbreeding of domestic animals with wild aurochsen (Bonfiglio et al., 2010). Taurine cattle reached the New World by European importations after the discovery of America in the late fifteenth century, and their descendants living today are broadly referred as Creole cattle.

Zebu cattle also spread around the world accompanying human migrations, but became more endemic in tropical and subtropical regions due to their adaptability to these environments. Zebu cattle were probably introduced in the African continent in the seventh century by imports of *B. indicus* sires during Arabian invasions (Bradley et al., 1996). Indian zebu cattle were only introduced in Central and South America in the early twentieth century, and started to be massively imported to the American continent after 1950.

### Formation of specialized breeds and further cattle adaptation

After domestication, farmers started to control cattle mating according to their interest in traits such as size, behavior, and milk production. This “breed the best to the best and hope for the best” strategy exerted a high artificial selective pressure, triggering a severe decline in cattle effective population size. Estimates from a genome-wide linkage disequilibrium analysis using a medium density SNP panel suggested that domestication was responsible for a 50 to 70-fold decline in the effective population size in comparison to the wild ancestors of taurine and zebu cattle (The Bovine HapMap Consortium, 2009).

About 200 years ago, farmers invented the concept of breed and started to limit germplasm exchange in order to standardize cattle populations based on morphology and performance, giving rise to over 1200 cataloged cattle breeds today (Taberlet et al., 2008; see also: http://www.ansi.okstate.edu/breeds/cattle/). In spite of this apparent rich source of genetic resources, over 200 additional breeds are already extinct, and many others are at risk (FAO, 2007).

Due to their high productive performance for milk and meat traits, some cattle breeds were adopted worldwide, such as the Holstein-Friesian, which is present in 128 countries (FAO, 2007). However, most of these specialized groups of genetically distinct animals are local, and even though they are not under the spotlights, they exhibit high adaption to their environments. Therefore, small local breeds should be considered as primary targets of SS studies, as several of them are endangered and their genomes shelter footprints of adaptation.

### Implications to positive selection mapping

In theory, neutrally evolving alleles form the majority of the genome variation within and between species, and events such as population contraction, expansion, migration, isolation and admixture are responsible for random drift of such alleles. While the demographic history of a population determines its neutral allele frequency spectrum, alleles that impact fitness, survival, reproduction, or traits of human interest are subjected to natural or artificial selection, such that their frequencies deviate from the distribution of neutral alleles. Therefore, mapping loci under selection implicates searching for outlier alleles that substantially differ from the genome background.

The idea of outlier loci is dependent upon knowledge about the frequency distribution of neutrally evolving alleles. As different populations have distinct demographic histories, their allele frequency spectrum also differ. Consequently, statistical methods designed to map loci under selection must be calibrated to either a demographic model for the population under study or to the empirical distribution of scores across the genome, assuming most of the genome is evolving under neutrality. As most of the events that shaped cattle diversity at the species and breed level are still obscure, methods that are robust across different demographic scenarios that take advantage of genome-wide scores to detect outlier loci are more appropriate to analyze cattle data. Nevertheless, one can use the demographic model for cattle differentiation, domestication and expansion provided in this section and combine with specific models for specialized cattle breeds to simulate the distribution under neutrality and compare with empirical data.

## Methods for detecting positive selection in the cattle genome

As proposed by Darwin and Wallace (1858), positive selection is the phenomenon where phenotypes that increase the likelihood of survival and reproduction (i.e., that increase an individual's fitness) become more prevalent in populations over time. In the context of the genome sequence, if a specific allele confers advantage, its carrier is more likely to thrive and leave more offspring than non-carriers, causing the haplotype containing that beneficial allele to spread quickly and increase in frequency in the population (Sabeti et al., 2002, 2006).

The majority of the genetic variation found within and between populations is deemed to have little or no effect on fitness, so that haplotype frequencies follow Hardy-Weinberg expectations (Kimura, 1968; Hellmann et al., 2003). Therefore, positive selection leaves distinctive tractable patterns of genetic variation that differ from the neutrally evolving background DNA sequence. These patterns are broadly referred as “signatures” or “footprints” of selection (Oleksyk et al., 2010).

In this section, we describe classes of signatures in terms of different signals. In each class, whenever convenient, we describe the basis of some popularly adopted methods to highlight their strengths and weaknesses for cattle studies. For a broader overview, we suggest the reader to consult other previously published reviews on the topic (Sabeti et al., 2006; Oleksyk et al., 2010; Vitti et al., 2013; Qanbari and Simianer, 2014). Additionally, we provide examples of studies reporting putative loci under selection in different cattle breeds.

### Local genetic diversity depression

At each generation, recombination shuffles and breaks haplotypes down, producing linkage equilibrium. When selection increases the frequency of an advantageous allele, neighbor variants “hitchhike” and rise in frequency together so quickly that recombination does not prevent linkage disequilibrium, causing an entire chromosome segment to lose diversity. Therefore, positive selection can be probed by searching for chromosome regions where heterozygosity is much lower than expected under neutrality (Oleksyk et al., 2008).

Ramey et al. (2013) scanned Illumina® BovineSNP50 (50 k) genotypes of over 6000 animals from 13 taurine and one zebu breed using an *ad-hoc* method to identify selective sweeps reaching fixation. Briefly, they considered a candidate locus under selection if at least five contiguous SNPs presenting a minor allele frequency (MAF) > 0.01 spanned a chromosome segment of 200 kb or more. Although the strategy was successful to find validated selective sweeps, such as the *POLL* locus that controls horn development (Georges et al., 1993), they showed that the SNP ascertainment bias of the 50 k assay incurred in false positives in breeds that are genetically distant from the SNP discovery breeds. This is not unexpected, as the influence of the 50 k bias on population genetics parameters, such as heterozygosity, genetic structure and differentiation, is well documented (Decker et al., 2009). Therefore, methods relying on heterozygosity are largely prone to type I errors generated from the SNP discovery process. Ideally, genotype data should be generated from less biased commercial arrays, such as the Illumina® BovineHD (HD), or from re-sequencing efforts.

Another approach to search for deficit of heterozygosity is the identification of islands of runs of homozygosity (ROH). As long stretches of consecutive homozygous genotypes indicate identical-by-descent haplotypes (Gibson et al., 2006; Lencz et al., 2007), ROH have been recently used to characterize genome-wide inbreeding in cattle (Purfield et al., 2012; Ferenčaković et al., 2013; Kim et al., 2013). However, as recombination is random, the distribution of ROH across samples is expected to be highly heterogeneous under neutrality, so genomic hotspots of ROH can be a signal of selective sweep (Curik et al., 2014). Interestingly, the length of the run is negatively correlated with the number of generations back to the selective pressure or inbreeding event (Howrigan et al., 2011), so ROH size can be used to date the age of the selective sweep.

Focusing on the identification of ROH islands produced by recent artificial selection in U.S. Holstein cattle, Kim et al. (2013) proposed a new statistic, namely *F*_*RL*_, that compares local homozygosity between a population under artificial selection and a control population. Briefly, for each SNP, animals are scored as 1 if the locus is encompassed by a ROH or 0 otherwise. Then, *F*_*L*_ is computed as the proportion of animals with scores equal to 1. Finally, *F*_*RL*_ is obtained as:

$$F_{RL}=\;\ln\left(\frac{F_{L(selected)}}{F_{L(control)}}\right)$$

All scores are standardized (i.e., subtracting each value by the average score and dividing by the standard deviation) to produce a distribution with mean zero and variance one. Extreme positive values of *F*_*RL*_ represent changes in allele frequency in the artificially selected population in comparison to the control group, and therefore reflect homozygosity attributable to recent inbreeding, drift or selection at the analyzed locus. As for the approach adopted by Ramey et al. (2013), SNP density and ascertainment bias are important confounders that can produce false positive ROH (Ferenčaković et al., 2013), so commercial SNP assays must be used with caution here.

Methods that search for local absence of heterozygosity are most powerful to detect haplotypes that hitchhiked to fixation. However, allowing for some heterozygosity can be of help to detect partial sweeps, i.e., haplotypes under ongoing selection that have not reached fixation yet. For a given bi-allelic site, let *n*_*REF*_ be the number of observed reference alleles, *n*_*ALT*_ the number of observed alternative alleles, and p and q their respective frequencies. The expected frequency of heterozygote genotypes under Hardy-Weinberg Equilibrium is given by:

$$H=2pq=\frac{2n_{REF}n_{ALT}}{{\left(n_{REF}+n_{ALT}\right)}^{2}}$$

This expectation can be easily extended for a chromosomal segment containing multiple bi-allelic sites as:

$$H=\frac{2\Sigma n_{REF}\Sigma n_{ALT}}{{\left(\Sigma n_{REF}+\Sigma n_{ALT}\right)}^{2}}$$

Rubin et al. (2010) proposed running sliding windows across the genome, calculating *H* for these windows and then standardizing the obtained values. The method named as *ZH*_*p*_ produces standard deviation scores, and extremely negative values represent chromosome windows where the regional diversity is substantially lower than the average genome diversity. Although the method has been successfully used to map candidate variants under selection in chickens (Rubin et al., 2010), dogs (Axelsson et al., 2013) and pigs (Rubin et al., 2012), no studies applying *ZH*_*p*_ to cattle data have been published to date.

### Change in the allele frequency spectrum

After a complete selective sweep (i.e., the haplotype containing the selected variant reaches fixation), new mutations slowly restore local diversity in the course of many generations. As mutations are generally rare and may take a large number of generations to drift to high frequency under neutral evolution, the local heterozygosity depression signal is deemed to remain robust for several generations after the selective pressure has occurred.

Newly acquired mutations or derived alleles (i.e., variants that differ from the original or ancestral allele) occur in lower frequency in comparison to ancestral alleles under neutrality, but when they arise within a selective sweep they will hitchhike to high frequency quickly in the selected population. Therefore, another class of signals that emerge after the depression in local diversity is the enrichment for moderate to high frequency derived alleles. Several methods have been proposed in this category, including Tajima's (1983), Fay and Wu's (2000), and Δ*DAF* (Grossman et al., 2010).

The limitation of the use of these methods in cattle data is that inference of ancestral alleles should be preferably performed by comparison of domesticated cattle genomes with wild type genomes. As these are not available, ancestral allele information for cattle SNP assays were derived from genotypes of outgroup species, such as Gaur (*Bos gaurus*), Buffalo (*Bubalus bubalis*) and Yak (*Bos grunniens*), which are assumed to descend from a common founder *Bovinae* species (Matukumalli et al., 2009; Utsunomiya et al., 2013). However, as not all SNP probes hybridize against the genomes of these outgroup species, ancestral allele information for cattle is incomplete. Future availability of genome assemblies for these outgroup species may help to better infer ancestral status for common SNPs.

One way to bypass the limitation of ancestral allele information is by using samples from several genetically distinct populations to estimate average allele frequencies that could represent the spectrum in the common ancestral population. In this approach, instead of searching for enrichment of high frequency derived or rare alleles, one may look for a shift in the allele frequency spectrum in one population in comparison to the average across populations. Stella et al. (2010) successfully applied the negative composite log-likelihood (*CLL*) approach, an extension of the composite likelihood ratio (*CLR*) test (Kim and Stephan, 2002; Nielsen et al., 2005) to 13 taurine, 4 zebu and 2 synthetic breeds, and reported several candidate loci under selection. These included *KIT* (mast/stem cell growth factor receptor gene), responsible for the piebald and color sidedness phenotype (Durkin et al., 2012), and *MC1R* (melanocortin 1 receptor gene), incriminated in the black/red coat color in Holstein and Angus (Klungland et al., 1995). These loci were further confirmed using whole genome sequence data of German Fleckvieh (Qanbari et al., 2014).

Briefly, *CLL* is computed for each SNP window as:

$$CLL=-\sum_{i\;=\;1}^{k}\ln\;\left[1-Pr\left(d<\;|p_{i}-{\bar{p}}_{i}|\;|\;\mu_{i}\right)\right]$$

Where, relative to SNP *i*, *d* is any random value from the theoretical distribution of allele frequencies with mean μ_*i*_ = *p*_*i*_, *p*_*i*_ is the reference allele frequency averaged across populations, and *p*_*i*_ is the allele frequency in the population being investigated. The theoretical distribution of allele frequencies can be modeled as a binomial or a normal approximation to the binomial distribution. As calculations are very straightforward and only require a dataset with multiple breeds instead of ancestral allele information, it is general enough for cattle SNP data.

Another extension of the *CLR* statistic was proposed by Chen et al. (2010), namely *XPCLR* (cross-population composite likelihood ratio test), which attempts to detect a shift in allele frequency in a target population in respect to a reference population. Lee et al. (2014) applied *XPCLR* to Holstein and Hanwoo whole genome sequence data and reported that the chromosome segment encompassing the kappa-casein gene (*CSN3*) exhibited high *XPCLR* scores in Holstein cattle.

### Long-range haplotypes

The concept of Extended Haplotype Homozygosity (*EHH*), first introduced by Sabeti et al. (2002), attempts to identify haplotypes that increased so rapidly in frequency that recombination could not substantially break them down, so linkage disequilibrium presents a long-range persistency. Briefly, consider *N* chromosomes in a sample, and *G* unique haplotypes extending from a core SNP site to an upstream or downstream position *x*, with each group *g* having *n*_*g*_ observations. The *EHH* score for the entire sample is calculated as:

$$EHH=\;\frac{\sum_{g\;=\;1}^{G}\left(\frac{n_{g}}{2}\right)}{\left(\frac{N}{2}\right)}$$

This score serves as a proxy for the probability of identity-by-descent of haplotypes within the chromosome segment being investigated. Generally, *EHH* is calculated at varying distances from the core SNP position, so that the decay of *EHH* as a function of physical distance can be assessed to determine the extension of the haplotype homozygosity. From this seminal concept, a family of statistical methods was developed in order to scan entire genomes in the search for evidence of selection.

Voight et al. (2006) proposed to measure how rapidly *EHH* decays from a core SNP site by calculating the area under the *EHH* curve,

$$iHH=\;\int_{a}^{b}EHH(x)dx$$

where *iHH* represents the definite integral of *EHH* evaluated over the domain of the chromosome segment delimited by upstream position *a* and downstream position *b* where *EHH* decays to some arbitrary small value (originally 0.05). As the area under the curve is not tractable analytically, a trapezoid quadrature with non-uniform grid can be adopted as a deterministic approximation:

$$iHH\sim \;\sum_{k\;=\;1}^{K}\left(x_{k\;+\;1}-x_{k}\right)\frac{\left(EHH_{k\;+\;1}+EHH_{k}\right)}{2}$$

A within population score, namely Integrated Haplotype Score (*iHS*), for a given site *i*, was introduced by Voight et al. (2006) as the log-ratio between the integrated *EHH* for the haplotypes containing the ancestral allele (*iHH*_*A*_) and the derived allele (*iHH*_*D*_):

$$iHS_{i}=\;\ln\;\left(\frac{iHH_{A,i}}{iHH_{D,i}}\right)$$

These scores are then standardized to have mean zero and variance one.

Extremely negative standardized *iHS* values have been of particular interest in human genetics, as they represent a recently acquired mutation that increased very rapidly in frequency (i.e., there is a partial sweep due to ongoing selection) or a haplotype that hitchhiked to fixation and then became enriched for derived alleles (Voight et al., 2006). However, a sweep can also produce large positive *iHS* values at nearby SNPs if ancestral alleles hitchhike with the selected site, so the chromosome region surrounding the selected variant typically exhibits a cluster of extreme positive and negative *iHS* values. Furthermore, in the context of cattle data, artificial selection and domestication probably favored “beneficial” alleles in the sense of human interest, regardless if it is ancestral or derived. Therefore, both positive and negative values should be investigated in cattle data. This implicates that the absolute value of standardized *iHS* scores should be preferred over the signed values, or, equivalently, that a two tailed hypothesis test should be assumed. As only partial ancestral allele information is available for cattle SNP assays, and the search for footprints of selection by *iHS* in cattle should disregard the direction of the sweep, a more appropriate generalized version of *iHS* can be postulated as the log-ratio between the integrated *EHH* for an arbitrary reference allele (*iHH*_*REF*_) and for the alternative allele (*iHH*_*ALT*_).

One of the limitations of this method is that if a given marker presents a nearly or completely fixed allele in the population being analyzed, this allele will have no integral to be calculated or an integral close to zero, so the log-ratio will result in a positive or negative infinite value. In this scenario, the calculation of *iHS* must be conditioned by *iHH*_*REF*_ > 0 and *iHH*_*ALT*_ > 0, which indirectly leads to a minor allele frequency (MAF) constraint. This limitation renders *iHS* underpowered to detect very recent nearly fixed selective sweeps, which are of primary interest in the cattle community. However, as discussed earlier, a crucial point to be considered is that contiguous chromosomal segments containing SNPs with MAF = 0 can also result from SNP chip ascertainment bias, which may produce false positive signals.

Tang et al. (2007) and Sabeti et al. (2007) have independently developed equivalent methods, *Rsb* and *XPEHH*, respectively, which attempt to compare long-range haplotypes between populations in order to increase the power of selective sweep detection. The most crucial improvement is that, for each population being analyzed *iHH* is calculated for the entire sample, instead of being partitioned between derived and ancestral alleles. This eliminates the MAF constraint and recovers the power to detect sweeps reaching fixation. The comparison with a population where the selective sweep may not have occurred adds extra power to the method. Calculations are performed as follows:

$$XPEHH_{i}=Rsb_{i}=\;\ln\left(\frac{iHH_{pop1,i}}{iHH_{pop2,i}}\right)$$

Where, relative to SNP *i*, *iHH*_*pop*1, *i*_ is the integrated *EHH* in the first population and *iHH*_*pop*2, *i*_ is the integrated *EHH* in the second population. Scores are also standardized to produce a distribution of standard deviates. Positive values indicate selective sweeps in the population used in the numerator, while negative values indicate selection in the population used in the denominator. Here, it is easy to keep track of the signals by using one-tailed hypothesis tests.

Studies applying *EHH*-based methods to cattle data are numerous (for instance, Hayes et al., 2008; Gautier and Naves, 2011; Qanbari et al., 2011, 2014; Flori et al., 2012; Utsunomiya et al., 2013; Huson et al., 2014). The reported loci are deemed to be genome responses to a variety of different selective pressures, such as milk and meat production, coat color, heat stress, and reproductive performance. Among these, one particularly interesting selective sweep, most likely related to adaptation to heat stress, has been reported in Creole cattle, including Senepol, Carora, Romosinuano, and cross-bred lineages (Flori et al., 2012; Huson et al., 2014). These cattle breeds present the slick hair coat phenotype, a dominant trait associated to heat tolerance in tropically adapted cattle that descend from Spanish cattle introduced to the New World. The chromosome segment containing the selective sweep ranges from 37.5 to 39.6 Mb on chromosome 20, with a variable peaking position (39.5 or 37.7 Mb) depending on the SNP panel (BovineSNP50 or BovineHD) and dataset analyzed (Flori et al., 2012; Huson et al., 2014). The disputed positional candidate genes are the retinoic acid induced 14 (*RAI14* or *NORPEG*), prolactin receptor (*PRLR*), and S-phase kinase-associated protein 2 (*SKP2*). A strong candidate mutation has been recently proposed for *PRLR*, a single base deletion in exon 10 (ss1067289408) predicted to cause a frameshift that introduces a premature stop codon (p.Leu462^*^) and consequent loss of 120 C-terminal amino acids from the long isoform of the prolactin receptor (Littlejohn et al., 2014).

### Population differentiation

Following the same principle as in *Rsb*/*XPEHH*, *XPCLR* and *F*_*RL*_, although positive selection may act across populations sharing geographical proximity, environmental factors or a common phenotype, outgroup populations may not share the same selective pressure. Therefore, changes in allele frequency promoted by selection in one group will not be detectable in the other, and large differences in allele frequencies between populations will be observed.

The fixation index *F*_*ST*_ (Wright, 1950; Weir and Cockerham, 1984) and its abundant estimators is the gold standard method for detecting highly differentiated loci between populations. In essence, given the average allele frequency *p* across subpopulations, *F*_*ST*_ is simply the ratio between the variance in the allele frequency in different subpopulations $\sigma_{S}^{2}=\sum_{j\;=\;1}^{k}{\left(p_{j}-\bar{p}\right)}^{2}$ and the variance in the total population σ^2^_*T*_ = *p*(1 − *p*). In pair-wise comparisons, calculations simplify to:

$$F_{ST}=\frac{{\left(p_{1}-p_{2}\right)}^{2}}{\left(p_{1}+p_{2}\right)(2-p_{1}-p_{2})}$$

Scores can be averaged across SNP windows or smoothed against genomic positions using a local variable bandwidth kernel estimator (Porto-Neto et al., 2013).

Flori et al. (2009) applied *F*_*ST*_ to three French dairy cattle breeds (Holstein, Normande, and Montbéliarde), and found that some of the putative loci under selection in that study overlapped genes that strongly affect milk production traits, such as the growth hormone receptor (*GHR*), and coat color, for instance *MC1R*. Porto-Neto et al. (2013) generated a comprehensive map of divergent loci between taurine and zebu cattle using over 777,000 SNPs and 13 cattle breeds, and reported that the highest scoring locus in the *F*_*ST*_ analysis maps to chromosome 7:47.2–53.7 Mb, which shelters a cluster of immune-related genes and *SPOCK1*, a gene previously implicated in puberty (Fortes et al., 2010).

Extensions of *F*_*ST*_ led to the development of the *FLK* test (Lewontin and Krakauer, 1973; Bonhomme et al., 2010; Fariello et al., 2013), which not only account for effective population size and hierarchical structure among populations, but also have known distributional properties under neutrality. Briefly, let *q*_*i*_ be the vector of reference allele frequencies at marker *i* for the populations being compared, and *q*_0_ the ancestral allele frequency for the same allele. The *FLK* method relies on the linear model:

$$q_{i}=1_{n}q_{0}+e$$

where the residual term *e* is assumed 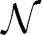
(0, *V*_*i*_), and *V*_*i*_ is the expected variance-covariance matrix for vector *q*_*i*_. This matrix is modeled as:

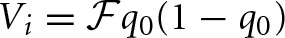



where 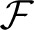 is a kinship matrix. The diagonal elements of 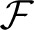 represent the expected inbreeding coefficients in each respective population, and off-diagonal elements represent the amount of drift accumulated on the different branches of the population tree. A weighted least squares estimate of *q*_0_ is then obtained as:

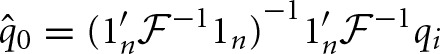



The *FLK* score is a measure of goodness-of-fit of this model, and is calculated as the deviance (i.e., residual sum of squares):

$$FLK_{i}={\left(q_{i}-{\hat{q}}_{0}1_{n}\right)}^{\prime }V_{i}{}^{-1}(q_{i}-{\hat{q}}_{0}1_{n})$$

Under the assumption of a star-like tree-pure drift model (i.e., the populations being compared evolved in parallel from a single ancestral population, with no mutations or admixture), matrix 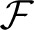 can be simplified to 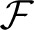 = *I*_*n*_*F*_*ST*_, where *I*_*n*_ is an identity matrix and *F*_*ST*_ is the average *F*_*ST*_ over all SNP loci. In this case, the average allele frequency across populations is an unbiased estimator of *q*_0_, and the deviance is simplified to *F*_*ST*_(*n* − 1)/*F*_*ST*_, which gives a test statistic that is linearly correlated with *F*_*ST*_. As discussed later, while *F*_*ST*_ has no known theoretical distribution under the neutral model, *FLK* can be modeled as a chi-squared distribution. Moreover, *FLK* outperformed *F*_*ST*_ in simulations, especially when scores were computed based on haplotypes (*hapFLK*) instead of single markers (Fariello et al., 2013). This method is a suitable alternative to *F*_*ST*_ for cattle data.

### Distributions under the null hyposthesis and *p*-values

Although different in formulation and assumptions, all methods presented so far attempt to address the same null and alternative hypothesis: *H*_0_ = locus is neutral; *H*_1_ = locus is not neutral. Under the hypothesis of neutrality, *EHH*-based methods are standard normal deviates (Voight et al., 2006; Sabeti et al., 2007; Tang et al., 2007). Hence, the probability that SNP *i* with *Rsb* or *XPEHH* score *x*_*i*_ is neutral in the population used as numerator can be approximated by an upper tail *p*-value derived from the normal cumulative density function (CDF) Φ:

$$Pr(Rsb,XPEHH>x_{i}\;|\;neutral)=1-\Phi \left(x_{i}\right)$$

Likewise, the probability that SNP *i* with *iHS* score *x*_*i*_ is neutral, given that both reference and alternative alleles are of interest, can be approximated by a two-tailed *p*-value:

$$\begin{array}{l} Pr(|iHS|>|x_{i}|\;|\;neutral)=1-|\Phi \left(x_{i}\right)-\Phi \left(-x_{i}\right)|\\ \;=1-2\left|\Phi \left(x_{i}\right)-0.5\right| \end{array}$$

Recently, concerns have emerged on the interpretation of *p*-values in signatures of selection analyses (Simianer et al., 2014). It is argued that, at least for the cases of *EHH*-based methods, scores standardized using genome-wide data are not test statistics in the classical sense but only deviates from an average (Voight et al., 2006), so *p*-values would only represent quantiles from an empirical distribution, rather than formal significance values. This implies that the probability of obtaining an arbitrary score given the locus is neutral cannot be exactly computed once the underlying true probability distribution may vary according to different demographic scenarios. The major caveat here is, for any given test discussed, the proposed asymptotic distribution under the hypothesis of neutrality is still largely based on coalescent simulations with demographic models calibrated for human data. However, if the majority of the genome is under neutrality and the null distribution is robust to a wide range of demographic models, one may expect that genome-wide distribution of scores in cattle data should mimic the simulations performed for the human model, serving as a control (Gianola et al., 2010). This has been observed in SS studies in cattle (Gautier and Naves, 2011; Flori et al., 2012; Utsunomiya et al., 2013), and therefore these theoretical distributions under neutrality are suitable for cattle data.

In the case of *F*_*ST*_, although approximate distributions are available (e.g., exponential or beta), such approximations are sub-optimal. One advantage of *FLK* over *F*_*ST*_ is that scores are asymptotically distributed as χ^2^_ν_ under the null hypothesis, where degrees of freedom ν can be equivalently calculated as *number of subpopulations* − 1 or as the average *FLK* across all loci. Upper tail *p*-values can be computed as:

$$Pr(FLK>x_{i}\;|\;neutral)=1-F_{\nu }(x_{i})$$

where *F*_ν_ is the χ^2^ CDF with ν degrees of freedom.

Although *F*_*ST*_ and *CLL* scores have unknown distributions under neutrality, both rely on population comparisons. The use of datasets consisting of multiple populations allows for permutation tests via random sampling of individuals or random sorting of population labels in order to compute an empirical null distribution. Nevertheless, permutation tests are computationally intensive and may be impracticable in re-sequencing data.

The *ZHp* method suffers from the same problem, with the additional limitation of not allowing data permutations when a single population is surveyed. Although a truncated normal distribution could be suggested to approximate its null distribution, this has not been properly assessed in practice. Another challenge is deriving significance values when scores are averaged across SNP windows. Following the assumption that the majority of the loci (single markers or SNP windows for that matter) are under neutrality, an empirical CDF derived from genome-wide scores should converge to the underlying true CDF, so probability values could be empirically obtained from a step function:

$$\begin{array}{l} Pr(Test>x_{i}\;|\;neutral)=1-ECDF\left(x_{i}\right)\\ \;=1-\frac{\left(number\;of\;scores\;\leq \;x_{i}\right)}{number\;of\;observed\;scores} \end{array}$$

The probability that SNP *i* with score *x*_*i*_ in a given test is selected is not as trivial to approximate. While the distributions under the hypothesis of neutrality seems to be robust across a wide range of demographic models, the distribution under the hypothesis of selection may vary widely depending upon the demographic history that shaped the allele frequency spectrum. Therefore, there is no unique theoretical distribution to represent selected variants, and coalescent simulations using well calibrated demographic models are required in order to generate empirical distributions. This has been successfully done for human data (Grossman et al., 2010), but is highly challenging in cattle as most of the demographic history of the bovine species is yet to be uncovered. Nevertheless, promising methods to infer demographic scenarios from the data, including estimates of population sizes and population separations over time, are now emerging (Schiffels and Durbin, 2014), which could be useful to elucidate cattle history and customize coalescent simulations based on empirical data.

### Available software and analysis best practices

Table 1 summarizes all the software mentioned below. The first step to be considered before starting a SS analysis is filtering out poor quality data and markers and samples that are not informative or that may confound the analysis. Discussing the particularities of measures of quality control is beyond the scope of the present article, but some of the best practices in SS studies can emerge from common sense. First, metrics such as Hardy-Weinberg Equilibrium (HWE) and MAF, highly used in GWA studies, should be applied with caution. Elimination of markers with extreme deviations from HWE expectations may counteract the whole SS enterprise, as in this type of study outlier loci are being sought. Likewise, MAF controls may cause signals that are reaching fixation to be completely lost. A situation where HWE and MAF thresholds can be benign is when only markers with extreme excess of heterozygotes are eliminated, and MAF constraints are applied to the pooled allele frequencies across all populations in the study. We suggest *PLINK* v1.07 (Purcell et al., 2007) or *PLINK* v1.90 for this first data screening. Other usual filters such as GenCall and GenTrain scores and call rates should follow the same guidelines as in GWA studies.

**Table 1:** **Available software for signatures of selection analysis**.

**Software**	**Analysis**	**Availability**	**References**
PLINK v1.07 and v1.90beta	Quality control, data management, cryptic relatedness check, and computation of runs of homozygosity and FST	http://pngu.mgh.harvard.edu/~purcell/plink/	Purcell et al., 2007
		https://www.cog-genomics.org/plink2	
SNP & Variation Suite v7.6.8	Quality control, data management, cryptic relatedness check, and computation of runs of homozygosity and FST	http://www.goldenhelix.com	Golden Helix, Bozeman, MT, USA
GCTA v1.24.2	Quality control, data management, and cryptic relatedness check	http://www.complextraitgenomics.com/software/gcta/	Yang et al., 2011
fastPHASE v1.2	Phasing and imputation of missing genotypes	http://stephenslab.uchicago.edu/software.html#fastphase)	Scheet and Stephens, 2006
Beagle v3.3 and v4.0	Phasing and imputation of missing genotypes	http://faculty.washington.edu/browning/beagle/beagle.html	Browning and Browning, 2008
SHAPEIT2	Phasing and imputation of missing genotypes	https://mathgen.stats.ox.ac.uk/genetics_software/shapeit/shapeit.html	O'Connell et al., 2014
Sweep	EHH-based methods	http://www.broadinstitute.org/mpg/sweep/	Sabeti et al., 2002
selscan	EHH-based methods	https://github.com/szpiech/selscan	Szpiech and Hernandez, 2014
rehh	EHH-based methods	http://cran.r-project.org/web/packages/rehh/index.html	Gautier and Vitalis, 2012
FLK	Computation of the FLK statistics	https://qgsp.jouy.inra.fr/index.php?option=com_content&view=article&id=50&Itemid=55	Bonhomme et al., 2010; Fariello et al., 2013
SweepFinder	Computation of the CLR statistics	http://people.binf.ku.dk/rasmus/webpage/sf.html	Nielsen et al., 2005
XP-CLR v1.0	Computation of the XPCLR statistics	http://genetics.med.harvard.edu/reich/Reich_Lab/Software.html	Chen et al., 2010
cosi and cosi2	Coalescent simulations	http://www.broadinstitute.org/~sfs/cosi/	Schaffner et al., 2005; Shlyakhter et al., 2014
		http://www.broadinstitute.org/~ilya/cosi2/	
MSMS	Coalescent simulations	http://www.mabs.at/ewing/msms/index.shtml	Ewing and Hermisson, 2010

Another important issue is cryptic relatedness. Eliminating samples of closely related animals is of paramount importance to reduce false positive signals. We have previously reported an algorithm to find the maximum independent set based on identity-by-descent, i.e., maximize the number of samples while eliminating first and second degree relationships using SNP data (Utsunomiya et al., 2013). Other software such as *PLINK* v1.90 and *GCTA* v1.24.2 (Yang et al., 2011) also provide means to find the optimal set of independent individuals.

The next key step is producing high quality phased data. There are several methods and implementations for phasing, such as *fastPHASE* v1.2 (Scheet and Stephens, 2006), *Beagle* v3.3 or later (Browning and Browning, 2008), and *SHAPEIT2* (O'Connell et al., 2014). Although *fastPHASE* exhibits high phasing accuracy, it is orders of magnitude more computationally intensive than *Beagle* or *SHAPEIT2*. It is important to notice that phasing is not a straightforward procedure, and is highly prone to errors. Consequently, results from haplotype-based methods should be assessed with caution. The effect of haplotype errors on SS results remains underexplored and at some extent neglected.

Regarding the SS analysis *per se*, *EHH*-based methods can be computed using *Sweep*, *selscan* (Szpiech and Hernandez, 2014) or the *R* package *rehh* (Gautier and Vitalis, 2012). *F*_*RL*_ has a dedicated software, as well as *CLR* (available at: and *XPCLR*. For ROH-based tests, runs can be computed using either *PLINK* or *SNP & Variation Suite v7.6.8* or later, and *F*_*RL*_can be easily computed with home grown scripts. In the cases of *F*_*ST*_, *CLL*, *ZH*_*p*_ and the method reported by Ramey et al. (2013), allele frequencies and genotype counts can be obtained from either *PLINK* or *SNP & Variation Suite*, and calculation of scores can be easily implemented in *R* (available at: http://www.r-project.org/) or other languages. Simulations of population genetics datasets under specific demographic models, including neutral and selected loci, can be performed using coalescent simulators such *cosi* (Schaffner et al., 2005), *cosi2* (Shlyakhter et al., 2014), or *MSMS* (Ewing and Hermisson, 2010).

## Combining selection signals

The available methodologies to detect positive selection differ substantially from each other in terms of the pattern of genetic variation encrypting a “signal.” However, all of them have a shared objective: to identify loci that have undergone positive selection. Indeed, at least for recent selective pressures (up to a few thousand generations back), a selected variant is expected to be in a chromosome segment where there has been loss of diversity, enrichment for derived or rare alleles, population differentiation, and highly frequent long-range haplotypes. Therefore, collecting evidence across different methodologies targeting distinct classes of signals may help in identifying loci under positive selection. This section explores the statistical properties and limitations of some of the available methods designed to combine different methods for signatures of selection.

### Composite of multiple signals (CMS)

In the original implementation of the method (Grossman et al., 2010), CMS is a local test designed to narrow down signals detected from *s* distinct genome-wide scans, and is defined as the approximate joint posterior probability that a given variant is selected:

$$\begin{array}{l} CMS_{local}=\mathrm{Pr}\left(selected\;|\;x_{ij}\right)\\ \;=\prod_{\text{j }=\;1}^{\text{s}}\frac{\mathrm{Pr}(x_{ij}|selected)\mathrm{Pr}(selected)}{\mathrm{Pr}(x_{ij}|selected)\mathrm{Pr}(selected)}\\ \;\;\;\;\;\;\;+\mathrm{Pr}(x_{ij}|neutral)\mathrm{Pr}(neutral) \end{array}$$

The genome-wide extension (Grossman et al., 2013) focuses on the product of the Bayes Factors for each one of the tests to be combined. For each test, BF is computed as the ratio between the posterior and prior odds:

$${\text{CMS}}_{\text{GW}}=\prod_{\text{j = 1}}^{\text{s}}{\text{BF}}_{\text{ij}}=\prod_{\text{j = 1}}^{\text{s}}\frac{\mathrm{Pr}(x_{ij}|selected)\mathrm{Pr}(selected)}{\mathrm{Pr}(x_{ij}|neutral)\mathrm{Pr}(neutral)}$$

In the absence of prior information on the number of loci under selection across the genome, CMS scores simplify to:

$${\text{CMS}}_{\text{GW}}=\prod_{\text{j = 1}}^{\text{s}}\frac{\mathrm{Pr}(x_{ij}|selected)}{\mathrm{Pr}(x_{ij}|neutral)}$$

A challenging aspect of the implementation of these methods is computing Pr(*x*_*ij*_|*selected*), which requires simulations under clear demographic assumptions. In contrast, as discussed earlier, many methods designed to detect markers under positive selection allow for approximating Pr(*x*_*ij*_|*neutral*) from asymptotic theoretical or empirical distributions. Therefore, composite tests considering only the distributions under neutrality are more appropriate for cattle data.

The original CMS score can be modified to take advantage of the assumed distributions under neutrality in order to relax demographic assumptions and avoid expensive simulations. The most essential modification involves reformulating the problem of detecting markers departing from neutrality. Instead of considering the assessment of whether a marker has been selected or not, one can look for support from the data against the null model, i.e., that the marker does not fit well to the neutral model. First, let the new CMS score be the approximate joint posterior probability of a given variant not being neutral:

$$\begin{array}{l} {\text{CMS}}_{\text{new}}=\mathrm{Pr}\left(not\;neutral|x_{ij}\right)\\ \;=\prod_{\text{j = 1}}^{\text{s}}\frac{\mathrm{Pr}(x_{ij}|not\;neutral)\mathrm{Pr}(not\;neutral)}{\mathrm{Pr}(x_{ij}|neutral)\mathrm{Pr}(neutral)}\\ \;+\mathrm{Pr}(x_{ij}|not\;neutral)\mathrm{Pr}\text{​}\left(not\;neutral\right) \end{array}$$

Here, Pr(*x*_*ij*_|*neutral*) is computed directly from its theoretical distribution, and Pr(*x*_*ij*_ | *not neutral*) is computed as 1 − Pr(*x*_*ij*_|*neutral*). Also, it can be assumed that the prior Pr(*not neutral*) = 1 − Pr(*neutral*). Therefore, the new CMS score can be re-written as:

$${\text{CMS}}_{\text{new}}=\prod_{\text{j = 1}}^{\text{s}}\frac{\left[1-\mathrm{Pr}\left(x_{ij}\text{​}|neutral\right)\right]\left[1-\mathrm{Pr}\text{​}\left(neutral\right)\right]}{\begin{array}{l} \mathrm{Pr}\left(x_{ij}\text{​​}|neutral\right)\mathrm{Pr}\text{​}\left(neutral\right)\\ \;\;\;\;+\left[1-\mathrm{Pr}\left(x_{ij}\text{​}|neutral\right)\right]\left[1-\mathrm{Pr}\left(neutral\right)\right] \end{array}}$$

Likewise, the genome-wide modified CMS scores can be reformulated as:

$${\text{CMS}}_{\text{GW }-\text{ new}}=\prod_{\text{j = 1}}^{\text{s}}\frac{\mathrm{Pr}(x_{ij}|not\;neutral)}{\mathrm{Pr}(x_{ij}|neutral)}=\prod_{\text{j = 1}}^{\text{s}}\frac{1-\mathrm{Pr}(x_{ij}|neutral)}{\mathrm{Pr}(x_{ij}|neutral)}$$

It is important to note that this modification does not allow for the same interpretation as the original CMS method: the composite likelihood does not indicate selection, but rather, that a marker does not fit well the neutral model.

### Meta analysis of selection signals (meta-SS)

Following the ideas expanded from the landmark publication of Grossman et al. (2010), given the probabilities under the null hypothesis for each test, our interest is to identify loci presenting consistent rejection of the neutral model across the different tests. For any given statistic, *p*-values are uniformly distributed in the interval between 0 and 1 under the null hypothesis. This property makes possible to use an inverse CDF, such as the Gaussian density, to produce scores for each test derived from a single theoretical distribution. Therefore, for each SNP *i* and test *j*, a new score can be computed as *Z*_*ij*_ = Φ^−1^(1 − *P*_*ij*_), where *P*_*ij*_ is the *p*-value. These Z-transformed *p*-values can be then averaged and standardized to produce a composite score.

We have previously described *meta* − *SS* (Utsunomiya et al., 2013), an abstraction of the Stouffer Z-transformation for combining different selection signals using the aforementioned framework. As the Stouffer method assumes the tests are uncorrelated under the shared null hypothesis and the use of pair-wise comparisons produce correlated scores, a weighted average was originally proposed to penalize dependent tests:

$$meta-SS=\;\frac{\sum_{j\;=\;1}^{s}\omega_{j}Z_{j}}{\sqrt{\sum_{j\;=\;1}^{k}\omega_{j}^{2}}}$$

where ω_*j*_ is the weight for test *j*. In this setting, a uniform penalization can be applied to control for the inflation of correlated tests. As this penalization does not incorporate the strength of correlations among tests, the *meta* − *SS* test can be modified to explicitly account for the magnitude of correlations between scores. Considering all scores are equally weighted, the corrected composite score can be computed as:

$$meta-SS=\;\frac{\sum_{j\;=\;1}^{s}Z_{j}}{\sqrt{k+2R}}$$

where *R* is the sum of all pairwise Pearson's product-moment correlations. Under the hypothesis of neutrality, these composite scores are distributed as *N*(0, 1), so the higher is the Z-transformed value, the worse the marker fits the neutral model. Upper tail *p*-values can then be obtained from the standard normal CDF.

Obvious limitations from *meta* − *SS* and CMS is the inability to incorporate statistics for which *p*-values cannot be derived. Randhawa et al. (2014) proposed Composite Selection Signals (CSS), a nonparametric interpretation of *meta* − *SS*, where fractional ranking is used instead of *p*-values to combine different tests. Briefly, the vector of test statistics for method *j* is first sorted and then ranked, taking values 1, …, *k*. Next, the vector of ranks is re-scaled by dividing all elements by *k* + 1, thus producing a variable ranging from 0 to 1. These re-scaled ranks are treated as they were *p*-values for the test statistics, and then combined as in the *meta* − *SS* approach. This strategy is equivalent to computing probabilities from an empirical CDF using a step function, as discussed earlier, which has an appealing feature: as fractional ranking can be generated for any particular test, signature blending is made feasible even if the theoretical distributions are unknown or if scores have been averaged in chromosome windows. However, a caveat is that the magnitude of the actual test statistics may be lost, so one may expect loss of power compared to the use of theoretical or simulated distributions.

### Principal components analysis

Simianer et al. (2014) proposed combining different tests by applying an eigendecomposition of the correlation matrix of the scores. The attractive feature of this method is the possibility of using standardized scores instead of approximate probabilities. However, as each principal component has heterogeneous loadings from each test, deriving a single synthetic score that summarizes all different tests remains a challenge in this framework.

## Challenges and future directions

In theory, genome-wide genotypes are a vast source of information that can be explored in the search for large effect mutations that underwent selection. However, the existing data and methods still suffer from power issues and confounding effects that can give rise to false positive and false negative signals.

Although simulations suggest that only marginal gains in power are obtained when the sample size is increased from tens to hundreds of unrelated samples, marker density and allele frequency spectrum seems to impact power dramatically (Lappalainen et al., 2010; Simianer et al., 2014). Genotypes derived from commercial SNP arrays have two important limitations in this context: (1) incomplete genome coverage by markers; and (2) ascertainment bias. The search for SS must be preferentially performed using high density SNP panels, although optimal average intermarker distances to detect a sweep may vary depending on the effective population size, extent of linkage disequilibrium and the nature of the signal. Regarding ascertainment bias, commercial SNP arrays are suitable for cattle populations that are closely related to the breeds used in the SNP discovery process, but there is no guarantee they will be informative in genetically distant populations. Indeed, with a few exceptions, little congruence has been reported between candidate selected loci identified using whole genome sequence and different commercial genotyping platforms in African humans not included in the HapMap data (Lachance and Tishkoff, 2013). Altogether, these arguments suggest that re-sequencing data is the optimal choice in SS studies in cattle. At some extent, the HD assay is appropriate, as it has a high-density coverage of the genome with SNPs that are less biased than competing panels.

Another important source of confounding comes from the methods available to detect SS. First, all methods assume that individuals have no recent relationships in their pedigrees, a condition that is hardly true and generally ignored. It is essential to filter the data for cryptic relationships and assure to include only samples that are unrelated for at least two generations. Second, most of the methods rely on haplotypes and SNP coordinates, so further improvement of phasing strategies and of the bovine reference genome assembly is crucial to assure high quality results. Third, variants can depart from neutrality not only due to positive selection, but also as consequence of demographic events such as bottlenecks, genetic drift and admixture. Distinguishing loci under selection from neutrally evolving loci remains a major challenge in the field, and will require refinement of existing methods and development of new tests. Nevertheless, combining signals across different methods seems to be a promising approach to mitigate the individual methodological limitations. Also, when available, the concomitant analysis of environmental data (e.g., temperature, humidity, precipitation, disease prevalence, etc.) may be of great help in distinguishing true positives and accelerating the link between signal and phenotype (Lv et al., 2014).

Well-planned study designs will be crucial to exploit the full potential of SS in the detection of large effect mutations favored by selection. The identification of common adaptive phenotypes, together with geographical information data, should be an important player in sampling and decisions of population comparisons. Cattle breeds that are not highly productive but that exhibit genetic local adaptation should be considered as priority targets, as their environmental fitness was probably forged by hundreds of years of natural and artificial selection. In the context of artificial selection for complex traits, as large cattle pedigree cohorts for genomic selection become available, it will be soon possible to actually assess rapid changes in allele frequency using historical data, rather than present date data only. First demonstrations of such ideas were presented by Decker et al. (2012), and are likely to be incorporated as routine monitoring tools of genomic resources in breeding programs in the future.

Recently, comparing candidate loci across GWA studies has been facilitated in cattle with the advent of the Animal QTLdb (Hu and Reecy, 2007). Similarly, results from a SS scan on the human 1000 Genomes data reported by Grossman et al. (2013) were made available through the CMS viewer tool (available at: http://www.broadinstitute.org/mpg/cmsviewer/). Pybus et al. (2014) have also launched a comprehensive database of SS in the 1000 Genomes data (available at: http://hsb.upf.edu/). The research community would highly benefit from the development of a SS database for livestock species, which would not only facilitate cross-referencing, but would also help researchers willing to dig deep into the functional meaning of the signals to select promising candidates emerging from multiple preexisting studies.

Finally, similarly to the recent developments in human SS (Kamberov et al., 2013), unraveling the functional relevance of the putative selected variants will demand interdisciplinary reasoning, compilation of a wide range of data types (e.g., transcriptomic, proteomic), and assemblage of an arsenal of *post-hoc* assays, such as genomic editing, culture, phenotyping and challenge of specific cell lines, production of knock-out models, and generation of cross-bred lines for confirmatory segregation analyses.

### Conflict of interest statement

The authors declare that the research was conducted in the absence of any commercial or financial relationships that could be construed as a potential conflict of interest.
